# Dural Lymphomas Unmasked: A Narrative Review of Extra-Axial Mimics and a Pragmatic Diagnostic Decision Algorithm

**DOI:** 10.3390/cancers18152369

**Published:** 2026-07-23

**Authors:** Luca Zavatto, Viviana Berti, Davide Costazza, Paolo Cipriano Cecchi, Andreas Schwarz, Alessandro Spimpolo, Mohsen Farsad, Matteo Bonatti, Andrea Bernardelli, Mauro Krampera, Carlo Visco, Pier Paolo Berti

**Affiliations:** 1 Department of Neurosurgery, Hospital of Bolzano (SABES-ASDAA), Teaching Hospital of Paracelsus Medical University (PMU), 39100 Bolzano-Bozen, Italy; luca.zavatto@sabes.it (L.Z.); davide.costazza@sabes.it (D.C.); paolocipriano.cecchi@sabes.it (P.C.C.); andreas.schwarz@sabes.it (A.S.); 2 Hematology and Bone Marrow Transplant Unit, Section of Biomedicine of Innovation, Department of Engineering for Innovative Medicine (DIMI), University of Verona, 37134 Verona, Italy; viviana.berti@univr.it (V.B.); mauro.krampera@univr.it (M.K.); carlo.visco@univr.it (C.V.); 3 Nuclear Medicine Unit, Hospital of Bolzano (SABES-ASDAA), Teaching Hospital of Paracelsus Medical University (PMU), 39100 Bolzano-Bozen, Italy; alessandro.spimpolo@sabes.it (A.S.); mohsen.farsad@sabes.it (M.F.); 4 Radiology Unit, Hospital of Bolzano (SABES-ASDAA), Teaching Hospital of Paracelsus Medical University (PMU), 39100 Bolzano-Bozen, Italy; matteo.bonatti@sabes.it; 5 Hematology and Bone Marrow Transplant Unit, General Medicine Department (DAI), Azienda Ospedaliera Universitaria Integrata di Verona, 37134 Verona, Italy; andrea.bernardelli@aovr.veneto.it

**Keywords:** dura mater, meningeal neoplasms, primary dural lymphoma, central nervous system neoplasms, meningioma diagnosis, differential magnetic resonance imaging, lymphoma, non-hodgkin

## Abstract

Dural-based lymphomas are rare extranodal non-Hodgkin lymphomas that span from indolent primary dural entities, most commonly the extranodal marginal zone (mucosa-associated lymphoid tissue) lymphoma, to aggressive variants that are primary or represent secondary involvement from systemic lymphoma. They overlap radiologically with benign meningiomas, dural metastases, and immune-mediated pachymeningitis, creating a risk of anchoring bias, delayed diagnosis, and unnecessarily extensive resections when limited tissue sampling would be sufficient for diagnosis. We conducted a structured narrative review to synthesize the epidemiology, clinico-pathological classification, imaging phenotypes, and management principles of dural lymphomatous disease. This study will especially focus on primary dural lymphoma (PDL) with additional discussion of secondary dural involvement. Emphasis is placed on the clinical value of a multiparametric diagnostic approach that integrates computed tomography contrast-enhanced magnetic resonance imaging with functional techniques, and fluorodeoxyglucose positron emission tomography/computed tomography for systemic staging and for distinguishing truly localized primary dural lymphoma from secondary involvement. Potential diagnostic pitfalls related to somatostatin receptor–based tracer uptake will also be discussed. We introduced a pragmatic operational framework based on four clinico-biological clusters, translated into a step-by-step decision algorithm that prioritizes timely biopsy and comprehensive hematologic staging to guide surgical strategy. Given the absence of dedicated, multidisciplinary guidance for dural-based lymphomas, this algorithm is intended as a reproducible foundation for consensus recommendations and future multicenter validation.

## Simple Summary

Primary dural lymphomas (PDLs) are uncommon extranodal non-Hodgkin lymphomas arising from dura mater. Most of the published guidance on their management is extrapolated from systemic lymphomas involving the central nervous system. On imaging, dural lymphomas may resemble benign meningiomas, metastases, or inflammatory disorders that thicken the dura. Accordingly, most patients initially undergo aggressive surgical intervention instead of limited diagnostic procedures. This review summarizes current knowledge on PDL, describes the most common disease subtypes (indolent histologies versus aggressive disease confined to the dura or secondary involvement), and highlights the value of a multidisciplinary, integrated approach combining conventional imaging with advanced magnetic resonance features, alongside neurosurgical, and onco-hematological expertise. We propose a pragmatic, hypothesis-generating step-by-step diagnostic approach to support timely and limited biopsy, staging, and treatment selection, with the aim of reducing misdiagnosis, avoiding unnecessary extensive surgery, and accelerating appropriate therapy.

## 1. Introduction

Dural-based lymphomas are rare but clinically relevant, ranging from (i) indolent primary dural lymphoma (PDL) —most commonly extranodal marginal zone lymphoma (EMZL)/mucosa-associated lymphoid tissue (MALT) lymphoma—to (ii) aggressive disease that may be primary dural (less often) or reflect dural involvement within secondary CNS lymphoma (SCNSL). These entities frequently share a “meningioma-like” radiological phenotype and therefore carry a non-negligible risk of delayed diagnosis and anchoring bias [1,2,3]. Quantitatively, a multi-institutional cohort of dural marginal zone lymphoma reported upfront tumor resection in 65% (17/26) of patients because the preoperative impression was meningioma [4]. Similarly, histopathology-confirmed cohorts confirm that dural MZL commonly presents as a dura-based enhancing mass with a meningioma-like phenotype [5], supporting the clinical relevance of a biopsy-first/biology-first strategy to reduce anchoring bias and unnecessary resections.

This distinction is not merely nosological but carries immediate management implications. When clinical and radiological red flags raise suspicion for a “meningioma-mimicking lesion” consistent with lymphoma, anchoring bias should be actively avoided and the diagnostic work-up promptly redirected towards histopathologic confirmation and appropriate staging.

Accurate classification (PDL EMZL/MALT, PDL non-EMZL/MALT including dural DLBCL, and secondary dural involvement/SCNSL) has direct consequences for the diagnostic work-up (extent of systemic staging and CSF assessment according to context), radiotherapy and surgical decision-making (biopsy vs. resection), the need for CNS-directed chemotherapy (particularly high-dose methotrexate [HD-MTX]-based regimens in aggressive cases), and follow-up strategies [6,7].

The 5th edition of the WHO classifications for CNS tumors and hematolymphoid neoplasms, together with international EHA–ESMO guidance for primary and secondary central nervous system lymphoma (PCNSL/SCNSL), provides the conceptual framework within which dural lymphomas should be positioned across the spectrum of CNS lymphoma and SCNSL [6,8,9,10]. However, these documents devote limited space to the specificities of pachymeningeal disease. Consequently, diagnostic and therapeutic strategies for dural lesions are often extrapolated from the far more abundant evidence on parenchymal presentations. At present, there are no dedicated guidelines for the management of dural lymphomatous lesions.

The primary aim of this narrative review, intended for a multidisciplinary neuro-oncological audience, is to summarize available evidence on epidemiology, imaging patterns (conventional and advanced MRI; 18F-FDG PET and, in selected settings, somatostatin receptor [SSTR] tracers), histopathological correlates, and management strategies for dural lymphomas, with particular emphasis on PDL. We further propose a pragmatic, hypothesis-generating, multidisciplinary operational framework comprising four clinico-biological clusters, along with a diagnostic pathway that integrates MRI and PET findings with hematologic staging, aimed at minimizing anchoring bias and avoiding unnecessary surgical resections.

## 2. Materials and Methods

### 2.1. Study Design and Objectives

Owing to the rarity of dural lymphomas and the heterogeneity of reported data—frequently nested within PCNSL or SCNSL series—we conducted a structured narrative review and critical synthesis with the following objectives: (1) define an operational nosological framework distinguishing PDL from secondary dural involvement/SCNSL; and (2) propose an integrated decision pathway—including hematologic staging—supporting differential diagnosis, therapeutic stratification, and treatment planning [1,3,8]. Within this framework, we emphasize multimodal imaging analysis, leveraging morphological and metabolic patterns to guide pre-biopsy diagnostic orientation in “meningioma-like” presentations and to support differential diagnosis against major extra-axial mimics.

### 2.2. Data Sources and Search Strategy

We searched PubMed/MEDLINE, Embase, and Scopus from database inception to November 2025, using combinations of free-text terms and, for PubMed, MeSH terms and keyword equivalents. Boolean operators (AND, OR, NOT) and truncation (*) were used to refine results. Search strings included, among others: dural lymphoma, primary dural lymphoma, dural marginal zone, MALT lymphoma dura, pachymeningeal lymphoma, secondary CNS lymphoma, meningioma mimics, diffusion, perfusion, FDG PET, and somatostatin receptor PET. The search was designed to map the evidence base and identify recurring themes (imaging and early management) rather than to produce quantitative estimates or pooled effect sizes. An example PubMed/MEDLINE search string is provided in the Supplement (Table S1).

### 2.3. Study Selection and Inclusion/Exclusion Criteria

We included: (1) observational studies (single-center or multicenter case series) reporting histotype, imaging, treatment, and outcomes; (2) reviews, position papers, and guidelines relevant to PCNSL/SCNSL and diagnostic–therapeutic frameworks; and (3) neuroradiological studies focused on imaging patterns and differential diagnosis of dural masses and/or CNS lymphoma. We excluded studies in which the lymphoma was predominantly intra-axial (parenchymal) and lacked a primary or dominant dural component; conference abstracts without full data; isolated case reports without additional management or radiological value; and non-English papers (except highly clinically relevant exceptions, documented).

### 2.4. Screening and Data Extraction

Two authors independently screened titles and abstracts and assessed full-text articles; disagreements were resolved by consensus. Extracted variables included: setting/denominator (PCNSL/SCNSL/systemic lymphoma cohorts), criteria for “dural-based disease,” histotype (EMZL/MALT vs. DLBCL vs. others), location (convexity/falx/skull base/spinal), MRI patterns (DWI/ADC; perfusion when available), PET (FDG ± SSTR), treatment (surgery/radiotherapy/chemotherapy), and outcomes (response, local/systemic relapse, and overall or progression-free survival when reported).

### 2.5. Methodological Quality Appraisal

To improve methodological transparency, we performed a structured methodological quality appraisal of the major included clinical studies, tailored to the predominant study designs (retrospective cohorts and case series). Because this work is a narrative review and not a meta-analysis, the appraisal was not used as a strict exclusion criterion. Instead, it was used to inform interpretive weighting of evidence, particularly for imaging-pattern descriptions, management inferences, and algorithm-supporting considerations.

For retrospective cohort studies, we assessed key risk-of-bias and reporting domains applicable to observational designs (e.g., clarity of cohort definition and denominator, ascertainment of dural involvement and histopathologic diagnosis, completeness of baseline imaging and staging description, treatment reporting, follow-up completeness, and outcome definition/reporting). For case series, we applied an analogous structured appraisal focusing on case selection, diagnostic ascertainment, consecutive inclusion (when stated), completeness of clinical/imaging characterization, intervention description, and outcome/follow-up reporting. Given the rarity of dural lymphoma and the limited literature base, studies were retained even when some domains were incompletely reported, but these limitations were considered in the narrative synthesis and in the weighting of conclusions.

Key appraisal domains across the major studies included in Table 1 are summarized in Supplementary Table S2.

### 2.6. Synthesis and Reporting

We organized the synthesis according to a biology-stratified framework: primary dural lymphoma (PDL; EMZL/MALT vs. non-EMZL/MALT) versus secondary dural involvement/SCNSL (aggressive vs. disseminated indolent lymphoma), focusing on practical consequences for diagnosis, staging, and treatment selection. Because the proposed algorithm is intended for front-end triage of “meningioma-like” dural-based lesions and prevention of anchoring bias at first presentation, we applied an explicit biology-stratified weighting of evidence when deriving imaging phenotype descriptions and initial decision nodes. Specifically, we prioritized PDL series (particularly cohorts in which dural mimicry and preoperative misclassification as meningioma were central clinical issues) to inform imaging phenotype characterization and biopsy-first decision logic. Evidence from cohorts with secondary dural involvement/SCNSL was not primarily used to infer meningioma-like dural mimic patterns. Instead, it chiefly informed (i) clinical red-flag triggers, (ii) systemic work-up and staging logic, and (iii) management principles after tissue diagnosis. Outcomes were summarized descriptively within operational clusters and were not pooled across PDL and secondary dural involvement/SCNSL populations. A summary table of key clinical series is provided in Table 1. Given heterogeneous designs and denominators (PDL series vs. cohorts derived from PCNSL/SCNSL), and the predominance of retrospective evidence, formal quantitative synthesis was not attempted. Instead, a structured methodological quality appraisal (Supplementary Table S2) was used to support interpretive weighting of evidence across studies, with particular attention to denominator clarity, diagnostic/staging ascertainment, completeness of imaging protocol reporting, and follow-up/outcome reporting.

## 3. Operational Definition and Classification

Dural involvement in CNS lymphoma lies between primary CNS lymphoma and the SCNSL spectrum, yet its rarity and exclusion from prospective studies often lead to its integration within broader categories (PCNSL sensu stricto or generic SCNSL), with potential for improper treatment. A clear operational definition of PDL versus secondary dural involvement is therefore critical for any diagnostic–therapeutic algorithm [3,6,8,9].

We use “primary dural lymphoma (PDL)” to denote a lymphomatous neoplasm arising from the pachymeninges in the absence of detectable systemic disease on standard hematologic staging (e.g., whole-body PET/CT and/or CT chest–abdomen–pelvis; ± bone marrow biopsy depending on context). Available series suggest that the most frequently reported histotype is dural EMZL/MALT, biologically akin to MALT lymphomas at other sites, typically “meningioma-like” on imaging, and generally associated with favorable outcomes with locoregional approaches [1,2,4,5,11].

In neuro-oncological cohorts drawn from PCNSL denominators, the histologic distribution may include a comparatively higher proportion of dural DLBCL, plausibly reflecting selection effects and referral bias [1].

Less commonly, PDL presents with non-EMZL/MALT histotypes, most notably intracranial or spinal dural DLBCL (PD-DLBCL) or follicular lymphoma and other variants. In these cases, although the lesion is dural-based, clinical behavior and treatment intensity resemble more closely aggressive CNS lymphoma [1,4].

Variations in terminology and case composition reflect differences in WHO classification frameworks. In brief, WHO CNS5 includes “MALT lymphoma of the dura” among primary CNS lymphomas and promotes an integrated, layered diagnostic approach based on morphology, immunophenotype, molecular/genetic data, and clinico-radiological context [8]. Conversely, WHO-HAEM5 considers dural EMZL/MALT a site-associated variant of MALT lymphomas, emphasizing its biological affinity with extra-CNS extranodal MALT [9]. Practically, these perspectives support a cautious, integrative approach combining neuro-oncology and hematology: (1) complete systemic staging per hematology standards, not only to exclude secondary CNS involvement, but also to identify synchronous or occult extranodal sites; and (2) prolonged follow-up, given the possibility—albeit uncommon—of late extracranial relapse [11].

“Secondary dural involvement” refers to pachymeningeal disease occurring in the context of known or concurrently diagnosed systemic lymphoma, i.e., within SCNSL. Here, the dura is one possible compartment of disease alongside brain parenchyma, leptomeninges, cranial nerves, and/or spinal roots; by definition, an extra-CNS component is detectable on systemic staging [3,7]. Most commonly, it reflects systemic DLBCL with CNS involvement at presentation or relapse, although scenarios in indolent and other aggressive lymphomas have been reported [3]. Clinical management follows SCNSL principles and is associated with an overall poorer prognosis compared with most PDLs [3,6,7].

In the remainder of this review, we use “dural lymphoma” as an umbrella term encompassing primary and secondary dural disease, structured into four operational subgroups: (1A) PDL, EMZL/MALT (“classic” indolent form); (1B) PDL, non-EMZL/MALT (predominantly PD-DLBCL; less often follicular lymphoma or other variants); (2A) dural involvement in SCNSL from aggressive lymphomas (predominantly DLBCL); and (2B) dural involvement in disseminated indolent lymphomas. These clusters are intended as biology- and management-oriented operational paradigms, not as an averaged representation of behavior across heterogeneous cohorts. In practice, the framework supports early differential diagnosis and staging decisions while keeping separate the evidence used to define meningioma-mimic imaging patterns (mainly PDL-based data) from the evidence guiding red-flag assessment, systemic staging, and post-diagnosis management intensity (primarily SCNSL/secondary involvement data).

Although simplified, this grid provides a shared multidisciplinary language and facilitates integrated interpretation of presentation, imaging, and therapeutic choices [1,3,4]. To facilitate an operational, bias-resistant interpretation of “meningioma-like” pachymeningeal lesions, Table 2 summarizes the typical clinico-radiological profiles of the four dural lymphoma clusters, highlighting key discriminators, early diagnostic priorities (biopsy and hematologic staging), and initial management implications. The schema reflects distinct clinico-biological and therapeutic paradigms and should not be interpreted as pooled or averaged behavior across heterogeneous PDL and SCNSL-derived cohorts.

## 4. Clinical Presentation, Epidemiology, and Therapeutic Overview

Although dural involvement is uncommon within the broader spectrum of CNS lymphomas, it carries significant clinical relevance due to the high risk of misclassification as a “meningioma-like” lesion and its resulting therapeutic implications. Misattribution to a benign extra-axial pathology may delay correct diagnosis and prompt inappropriate treatment strategies, in a setting where management differs substantially between indolent, predominantly locoregional disease and aggressive forms, whether primary or secondary.

Epidemiologic estimates are heterogeneous and strongly dependent on the denominator and referral patterns. One review suggested that PDL accounts for <1% of all CNS lymphomas; conversely, in PCNSL-based cohorts the proportion may appear higher, reaching 6.3% in a bi-institutional series (20/316 PCNSL) [1,2].

### 4.1. A. PDL, EMZL/MALT

Historical series identify dural EMZL/MALT as the prototypical PDL presentation. In the series by Venkataraman et al. (32 cases), there was a female predominance (27F/5M) and a median age of approximately 50 years [5]. Patients most commonly present with mass-effect symptoms from an extra-axial tumor (headache, seizures, focal deficits), with lesions on the convexity/falx/tentorium or skull base frequently interpreted preoperatively as meningiomas [4,11]

In terms of natural history, disease is often confined to the dura at diagnosis, with excellent responses after locoregional treatment (surgery and/or focal radiotherapy) [4,11]. Nonetheless, late extracranial relapse after complete local response has been described; this may reflect occult disease not detected at baseline and/or subsequent dissemination rather than “metastasis” in a strict sense [11].

Histologic confirmation is crucial because it directly informs prognosis and management: dural EMZL/MALT, typically indolent and frequently localized, is associated with more favorable outcomes than high-grade primary CNS lymphomas. Dural EMZL/MALT typically shows positivity for B-cell markers and BCL2, negativity for CD10 and BCL6, usually lacking MUM1 expression, with low Ki-67 expression [5]. A potentially helpful feature is the presence of clonal IgG4-positive plasma cells, documented by light-chain restriction (predominant κ or λ), a pattern described in marginal zone lymphoma involving the CNS. Importantly, IgG4 positivity per se does not equate to IgG4-related disease (IgG4-RD); some EMZL/MALT lymphomas may arise in an IgG4-RD background, and an etiopathogenetic relationship has been hypothesized [22].

In patients with EMZL/MALT lymphoma associated with hepatitis C virus (HCV) infection, initial antiviral therapy is recommended and may improve outcomes and, in select cases, induce lymphoma regression. Screening includes anti-HCV antibodies and, if positive, HCV-RNA PCR to confirm active infection and quantify viral load [23]. Direct-acting antivirals (DAAs) have transformed HCV treatment, achieving very high rates of sustained virologic response across most genotypes [24].

Beyond radiotherapy, systemic approaches—including HD-MTX–based and non–HD-MTX regimens—have been reported [25]. However, combined chemo-radiotherapy carries a substantial risk of neurotoxicity and is therefore generally unnecessary and not routinely recommended in this indolent, often localized setting [26].

### 4.2. B. PDL, Non-EMZL/MALT (DLBCL and Other Histotypes)

Non-EMZL/MALT PDL is uncommon in traditional dural pathology series, yet it may be relatively overrepresented in cohorts from tertiary neuro-oncology centers. In the series by Karschnia et al., DLBCL comprised 10 of 20 PDL cases, whereas MZL accounted for 6 of 20 [1]. Taken together, these data suggest that “MZL predominance” can coexist with a clinically meaningful DLBCL subset—an apparent discrepancy likely driven by differing denominators, referral bias, and center-specific case mix.

In immunocompetent patients, the immunophenotype typical of PCNSL-DLBCL reflects mature B-cell origin with post-germinal-center activation and a predominantly non-GCB (activated B-cell-like) profile: CD20+, CD79a+, PAX5+, usually CD10−, variably BCL6+, strong MUM1/IRF4+, high Ki-67 (>80–90%), and typically negative for CD5/CD138 in classic immunocompetent cases; an ABC/non-GCB phenotype is prevalent [27].

Clinically, PD-DLBCL shares a dural-based extra-axial localization and “meningioma-like” appearance with EMZL but tends to exhibit faster growth and neurological deterioration over weeks. Skull-base/cavernous sinus involvement with cranial neuropathies and spinal dural/epidural masses causing compressive myelopathy or radiculopathy have been reported [2,22].

Biologically and clinically, these forms often require systemic CNS-directed therapy analogous to aggressive PCNSL [1,6,10]. Combinations of HD-MTX with other cytotoxic agents capable of crossing the blood–brain barrier are recommended (ideally evaluated in prospective trials). Regimens cited include MATRix, R-MBVP, rituximab–HD-MTX–carmustine–etoposide–prednisone, R-MPV, and R-MT. High-dose chemotherapy followed by autologous hematopoietic stem cell transplantation (HDC-ASCT) is recommended as consolidation in eligible patients with responsive or stable disease after adequate induction therapy [6].

Beyond DLBCL, other PDL histotypes have been described (e.g., dural follicular lymphoma, mantle cell lymphoma with dural infiltration, and very rare dural presentations of other lymphoproliferative neoplasms), largely as case reports or small series. For these variants, treatment is necessarily extrapolated from experience with dural EMZL, PCNSL, and systemic lymphomas of the corresponding histotype [28,29,30,31,32]. Overall, compared with dural EMZL/MALT, these variants tend to exhibit more aggressive biology and less favorable outcomes.

### 4.3. A. Dural Involvement in SCNSL from Aggressive Lymphomas

Within SCNSL, dural disease typically occurs as part of a multicompartment pattern involving brain parenchyma, leptomeninges, and/or neuroaxial structures such as cranial nerves and spinal roots; isolated dural disease is uncommon. In unselected cohorts of systemic DLBCL, CNS involvement is overall infrequent and limited to a minority of patients; however, risk is heterogeneous and increases substantially in the presence of unfavorable clinical and/or biological features, including high-risk extranodal sites, high disease burden, and aggressive molecular profiles (e.g., lymphomas arising in immune-privileged sites, the MCD molecular class per LymphGen, and double-hit/double-expressor phenotypes), reaching approximately 10–15% in high risk patients [3,33,34].

SCNSL events typically arise early in the disease course—often within the first year—suggesting that a subset of patients may already harbor subclinical CNS involvement at baseline. These events are associated with a poor prognosis despite intensive treatment strategies [3,14]. Evidence informing prognostication, contemporary intensive strategies (including transplant-based regimens), neuroradiological MRI phenotypes, and extranodal marginal zone lymphoma cohorts with CNS/dural involvement is derived from retrospective series, prospective trials, and real-world datasets [12,13,15,16,17,18,19,20,35].

### 4.4. B. Dural Involvement in Disseminated Indolent Lymphomas

CNS involvement in indolent lymphomas is rare. In a large cohort the reported frequency was 2.8% (33/1163) [36]. In series focusing on indolent lymphomas with CNS involvement, the dural compartment may be under-represented or not consistently separated radiologically. Therefore, practical classification often relies on integrating neuroimaging with systemic staging [21]. Imaging may show solitary or multiple dural “meningioma-like” masses or diffuse pachymeningeal thickening, noting that mixed patterns with leptomeningeal/parenchymal components are not uncommon [21].

Treatment is guided by histology and systemic disease burden. Published cases of indolent B-cell lymphomas with dural/CNS involvement report rituximab-based systemic approaches, including bendamustine–rituximab (BR) and R-CHOP [37,38]. BTK inhibitors (e.g., ibrutinib) have also been used in selected scenarios [39].

## 5. Imaging

**Figure 1:**
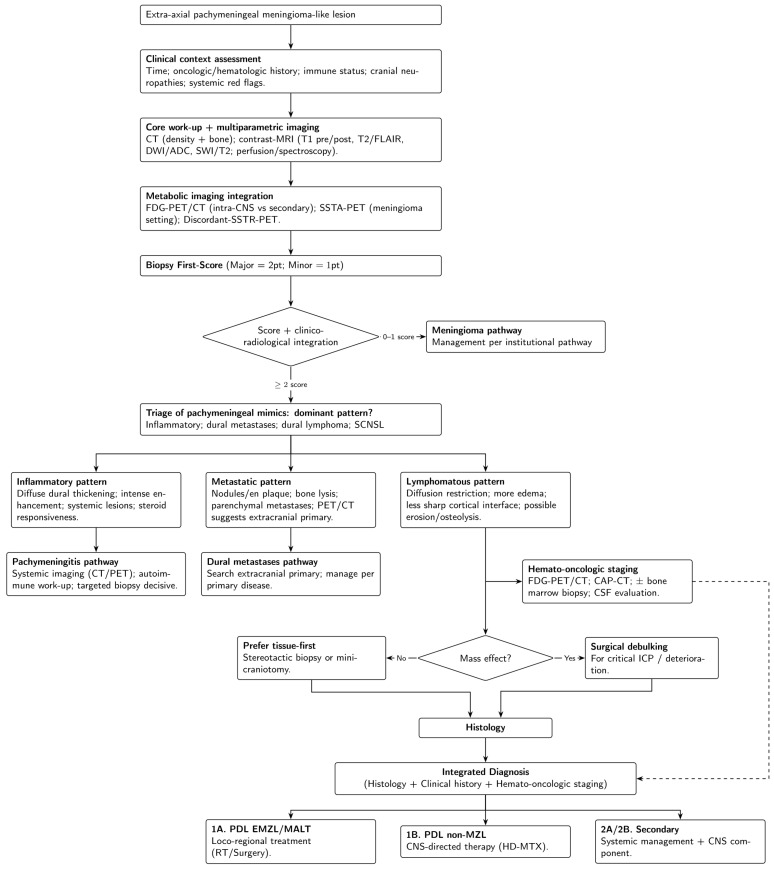
The algorithm applies to a “meningioma-like” extra-axial pachymeningeal lesion and proceeds through five main steps.
Clinical context assessment and acquisition of core imaging: CT (density and, crucially, bone changes: hyperostosis vs. erosion/osteolysis) plus contrast-enhanced MRI (pre-/post-contrast T1, T2, FLAIR, DWI/ADC, SWI/T2*; perfusion if feasible; ± spectroscopy/high-resolution 3D).PET/CT integration and Biopsy-First Score: FDG PET/CT to assess extra-CNS disease and support staging; SSTR PET in the meningioma setting with explicit attention to “discordant SSTR-PET” (SSTR uptake with red-flag CT/MRI and/or clinical features, such as osteolysis/erosion rather than hyperostosis, infiltrative/diffuse or multifocal pachymeningeal spread, marked diffusion restriction, disproportionate edema, rapid interval growth, or hematologic history/immunosuppression).Biopsy-First Score (Box 1) and initial branching: assign 2 points to Major criteria and 1 point to Minor criteria; apply pragmatic thresholds—0–1 low suspicion (standard pathway), 2–3 intermediate suspicion (biopsy-first favored if no decompressive indication), ≥4 high suspicion (biopsy-first plus early systemic staging).Pattern-based triage: if the overall profile is meningioma-typical (i.e., a coherent pattern across CT/MRI/PET suggesting a benign meningothelial lesion), follow the standard dural mass management pathway. Otherwise, triage major pachymeningeal mimics according to the dominant combined imaging/clinical pattern:
-Inflammatory: diffuse/multifocal thickening with intense enhancement; possible systemic inflammatory lesions; biopsy is often decisive.-Metastatic: nodular/en plaque/”carpet-like” disease; bone lysis; possible parenchymal metastases; PET/CT may suggest an extracranial primary.-Lymphomatous: meningioma-like dural mass with a supportive multiparametric profile (e.g., diffusion restriction and infiltrative interfaces, disproportionate edema, bone erosion/osteolysis, and/or discordant SSTR-PET), prompting tissue confirmation.Lymphomatous pattern: perform hematologic staging (whole-body PET/CT and/or CT chest–abdomen–pelvis; ±bone marrow biopsy; CSF assessment per clinical context) + define surgical strategy. In the absence of a primary decompressive indication (critical mass effect or medically refractory seizures), prioritize minimally invasive tissue diagnosis, reserve debulking for selected emergencies. After tissue diagnosis, integrate it with clinical history and systemic staging to assign the operational cluster (1A/1B/2A/2B) and corresponding therapy.

Imaging is central to the diagnostic–therapeutic pathway in CNS neoplasms, enabling non-invasive characterization and longitudinal monitoring through morphological, functional, and metabolic parameters. In dural lymphomas—where appearances can be nonspecific and mimic meningioma, pachymeningitis, or dural metastases—integrating CT, multiparametric MRI, and PET/CT is pivotal to strengthen suspicion, guide targeted biopsy, and help distinguish primary from secondary disease within staging workflows [40,41]. Management implications of imaging–staging integration and criteria prompting tissue sampling are addressed in “Future directions” (Box 1 and Box 2; Figure 1).

Evidence on CNS lymphoma imaging supports a shift toward multiparametric assessment beyond conventional sequences, incorporating diffusion, perfusion, spectroscopy, and PET (including hybrid PET/CT or PET/MRI). This aligns with WHO integrated diagnostics and major international recommendations [41,42]. However, most quantitative DWI/ADC and perfusion data derive from parenchymal PCNSL; applying these metrics to dural-based lesions is partly conceptual and requires caution, given overlap with atypical/anaplastic meningiomas and other “meningioma mimics.” Advanced parameters should therefore be interpreted alongside morphology, bone changes, growth pattern, and clinical context, rather than as stand-alone criteria [43,44].

A systematic approach is not merely technical quality control but a methodological requirement: reproducible MRI and PET/CT protocols share terminology among neuroradiology, neurosurgery, hematology, and oncology. A stable conceptual framework (here, the four clinico-biological clusters) is a prerequisite for using imaging as a stratification tool rather than a static depiction of the lesion.

This figure is intended as a hypothesis-generating triage framework for multidisciplinary decision support and not as a validated clinical decision rule.

### 5.1. Diagnostic Modalities: CT, MRI, and PET/CT

In suspected dural lymphoma, the imaging work-up is often sequential: initial cranial CT, followed by contrast-enhanced MRI and, when indicated, PET/CT (or PET/MRI) to define systemic extent.

CT remains essential as a first-line choice of imaging. In CNS lymphomas, including dural forms, lesions may appear iso- to relatively hyperdense and show intense, relatively homogeneous enhancement after iodinated contrast [40]. For dural masses, CT is particularly valuable for bone assessment: lymphoma more often shows a normal calvarium or erosive/osteolytic changes, whereas frank hyperostosis is less typical than in meningioma. Bone lysis should broaden the differential diagnosis to include lymphoma and metastasis [44,45].

Contrast-enhanced MRI remains the modality of choice for dural evaluation. A standardized protocol should include at a minimum, pre- and post-contrast T1, T2, FLAIR, DWI with ADC maps, SWI/T2*, and, when feasible, perfusion spectroscopy and high-resolution 3D acquisitions may be added [41]. MRI enables: (1) confirmation of extra-axial origin and mapping of dural extent; (2) assessment of relationships with parenchyma, leptomeninges, venous sinuses, and bone; (3) functional characterization via diffusion and perfusion [40,42].

18F-FDG PET/CT is particularly useful in hematology to identify extra-CNS sites and distinguish truly confined PDL from secondary disease with systemic involvement; it may also support targeted biopsy and response monitoring [41,46]. A separate consideration is SSTR PET (e.g., 68Ga-DOTATATE/DOTATOC), widely used in meningioma. When adjacent calvarial involvement is present, bone tracer uptake on SSTR PET may support the diagnosis of transosseous meningioma and help differentiate it from lymphoma-related osteolysis [47]. However, SSTR uptake should not be interpreted as a rule-in test for meningioma when discordant features are present [48]. We define “discordant SSTR-PET” as SSTR uptake compatible with meningioma but accompanied by a red-flag profile on MRI/CT and/or clinical assessment (e.g., erosion/osteolysis rather than hyperostosis, marked diffusion restriction, infiltrative or diffuse/multifocal pachymeningeal spread, disproportionate edema, multifocality or rapid progression, hematologic history or clinically significant immunosuppression) [49,50]. In these situations, the pathway favors a biopsy-first approach rather than proceeding directly to extensive resection [51].

### 5.2. MRI Sequences: Key Features and Practical Interpretation

Moving from modalities to sequences, MRI interpretation of dural lymphoma relies on combinations of findings rather than isolated signs.

Post-gadolinium T1 sequences define enhancement patterns: dural lymphomas frequently show intense, relatively homogeneous enhancement of a dural-based extra-axial mass, often with a “dural tail,” a nonspecific feature that contributes to confusion with meningioma. Some series report relatively extensive vasogenic edema, a less sharp cortical interface, and possible erosive bone changes [44,45].

T2 and FLAIR sequences, while less specific, help characterize edema and possible leptomeningeal involvement. Dural lymphomas are often iso- to moderately hyperintense on T2; cortico-subcortical edema may be more evident in biologically aggressive or secondary forms, and FLAIR facilitates detection of leptomeningeal extension beyond contiguity effects [40,41].

Diffusion (DWI) and ADC maps are among the most useful parameters to raise suspicion for lymphoma, but they are not pathognomonic in dural masses. Hypercellularity and a high nuclear-to-cytoplasmic ratio often produce diffusion restriction and low ADC values; however, restriction can also be seen in atypical/malignant meningiomas and other highly cellular dural tumors. Diffusion should therefore be interpreted alongside perfusion and morphology [40,43,44].

Only a limited number of studies have specifically investigated diffusion-weighted imaging in the assessment of dural lymphoma, and no validated ADC cut-off values are currently available to reliably differentiate dural lymphoma from its mimickers. Published case reports and small case series suggest that diffusion restriction is often homogeneous and may be more pronounced than in adjacent brain parenchyma; moreover, restriction appears more marked in DLBCL/SCNSL compared with EMZL/MALT [52,53,54,55,56]. A systematic evaluation of diffusion restriction patterns and quantitative ADC metrics in dural lymphoma represents, therefore, a promising area for future research.

MRI perfusion (DSC/DCE) may provide additional information. Typical meningiomas often show higher perfusion than many mimics, including some lymphomas, but exceptions exist and universally validated cut-offs are lacking. rCBV and related parameters should therefore be considered supportive markers within a multiparametric approach (morphology, bone involvement, DWI/ADC, and clinical context). Reported perfusion metrics vary across studies because of heterogeneity in acquisition and post-processing [44,57,58].

SWI/T2* can aid differential diagnosis: dural lymphomas typically show little or no intralesional susceptibility, consistent with limited hemorrhagic or calcified components, whereas susceptibility foci are more frequent in some dural metastases and in calcified meningiomas [42,44].

MR spectroscopy (MRS) contributes metabolic characterization, typically showing elevated choline with reduced N-acetylaspartate and possible lipid/lactate components in highly cellular lesions, including lymphomas. In meningiomas, an alanine peak is often described and may add supportive value [40]. Magnetic resonance spectroscopy of dural-based lesions remains technically challenging: susceptibility-related field inhomogeneities near bone and air-containing cavities can result in suboptimal shimming, and lipid signals from adjacent scalp and diploic marrow may contaminate spectra, limiting reliability and reproducibility [59].

Although diffusion restriction/low ADC, low-perfusion patterns, MR spectroscopy features, and PET findings may each increase suspicion for lymphoma in the appropriate context, substantial overlap exists between dural lymphoma, meningioma (including atypical/aggressive variants), metastases, and inflammatory pachymeningeal disorders. Therefore, no single imaging biomarker should be used in isolation as a rule-in criterion for dural lymphoma or meningioma. Imaging biomarkers should instead be interpreted as probabilistic, context-dependent features within a multiparametric and multidisciplinary diagnostic workflow.

### 5.3. Cluster-Based Analysis: Radiological Correlations

The practical value of these techniques emerges when applied to the four clinico-biological clusters. The goal is not to assign histology from imaging alone, but to use radiological information to strengthen or weaken the likelihood of each cluster in an individual patient.

In cluster 1A (PDL, EMZL/MALT), the typical scenario is an immunocompetent middle-aged woman with one or more “meningioma-like” extra-axial masses. MRI often shows iso- to hypointense T1 and iso- to moderately hyperintense T2 signal, homogeneous enhancement with a dural tail, and usually normal or minimally altered adjacent bone. Diffusion restriction may be present but not necessarily marked; perfusion and spectroscopy, when available, may support a less aggressive profile than dural DLBCL while remaining within the lymphomatous spectrum. FDG PET/CT is negative outside the dural lesion, and negative systemic staging supports an indolent PDL classification.

In cluster 1B (PDL, non-EMZL/MALT; especially PD-DLBCL), the initial appearance may be similar but with stronger aggressiveness signals: faster growth, more extensive vasogenic edema, a less sharp parenchymal interface, and more marked diffusion restriction with low ADC values. Bone erosion/osteolysis or more invasive involvement of the cortical plane may occur compared with typical meningioma [44,45]. FDG uptake may be high at the dural lesion while systemic staging remains negative; this combination favors cluster 1B.

Cluster 2A (dural involvement in SCNSL from aggressive lymphoma) is suggested primarily by the fact that the dura is rarely the sole compartment involved. Imaging may show a multicompartment pattern (parenchymal lesions, leptomeningeal involvement, diffuse or nodular dural thickening), and PET/CT may reveal systemic sites. Here, MRI characterizes CNS compartments, whereas integrated staging—often via PET/CT—defines systemic extent and biology [41].

Finally, cluster 2B (dural involvement in disseminated indolent lymphoma) can be radiologically similar to PDL MALT, with solitary or multiple “meningioma-like” dural masses, sometimes en plaque. In this setting, the discriminator is typically systemic context and staging: brain imaging alone may not reliably distinguish 1A from 2B, and classification depends largely on evidence of systemic disease [21,41].

### 5.4. Radiological Differential Diagnosis and Reasoning Schemes

In routine neuroradiological practice, many dural-based lesions are initially interpreted as meningiomas. However, a subset of “meningioma-like” masses represent mimics (metastatic, inflammatory, or lymphomatous) with substantially different management implications. To reduce anchoring bias, we propose a set of red flags (Box 1) and a decision pathway that prompts early tissue diagnosis and staging when appropriate.

Radiological differential diagnosis requires a multiparametric approach integrating extra-axial features, bone changes, enhancement patterns, and functional sequences (DWI/ADC and perfusion), acknowledging that no single sign is definitive and that advanced parameters are typically supportive within dural lesion assessment [43,44,60]. In selected contexts, a peripheral rim-like enhancement pattern may provide supportive value in distinguishing meningiomas from malignant dural tumors [61].

Usability improves when imaging findings are interpreted as combined patterns rather than as isolated signs. Accordingly, we frame the differential around dominant clinico-radiological patterns (meningioma-typical, inflammatory, metastatic, and lymphomatous) and align these patterns with the branch points of Figure 1 to mirror real-world neuroradiology and multidisciplinary board reasoning.

Dural metastases are an important source of diagnostic confusion: they can appear as solitary or multiple nodules, en plaque thickening, or diffuse “carpet-like” enhancement. Bone lysis (more than hyperostosis), coexisting parenchymal metastases, and PET/CT findings suggesting an extracranial primary would support this diagnosis; perfusion may help, but overlaps exist and vary by histotype [44,57,58].

Immune-mediated pachymeningitis (including IgG4-related forms) and neurosarcoidosis represent major inflammatory considerations in the differential diagnosis. MRI often shows diffuse or multifocal dural thickening with intense enhancement, sometimes symmetric; marked diffusion restriction is less typical, and perfusion tends not to show profiles strongly suggestive of a hypercellular neoplasm. Clinical context remains central: association with systemic manifestations (orbit, paranasal sinuses, salivary glands, lung parenchyma) and steroid responsiveness can be informative; nevertheless, targeted biopsy is often decisive [62]. In IgG4-related forms, serum IgG4 and inflammatory markers may support suspicion; on tissue, distinguishing a polyclonal from clonal plasma cell infiltrate through κ/λ light-chain restriction is critical and may indicate an underlying lymphoproliferative process (e.g., IgG4 + MZL).

### 5.5. Future Directions and a Pragmatic Management Proposal in the Absence of Dedicated Guidelines

In a field lacking specific recommendations, particularly for PDL, developing and validating shared approaches to the initial management of “meningioma-like” dural-based lesions is a priority. The goal is twofold: to reduce anchoring bias and standardizing clinically decisive choices (tissue diagnosis, timing and extent of staging, extent of surgery, and peri-diagnostic corticosteroid use). In practice, phenotypic overlap between meningioma and mimics (metastatic, inflammatory, or lymphomatous) and the inherently probabilistic nature of advanced imaging markers contribute to meaningful inter-operator variability, potentially affecting time to diagnosis, staging appropriateness, and selection of the initial surgical procedure. Figure 1 shows a reproducible decision pathway integrating key clinico-radiological nodes and corresponding actions (targeted biopsy, systemic staging, role of surgery, corticosteroid management) into a pragmatic sequence shared across disciplines. The pathway shown in Figure 1 (integrating Box 1 and Box 2) is intended as a pragmatic, hypothesis-generating triage framework to structure multidisciplinary discussion and reduce anchoring bias in the initial assessment of “meningioma-like” dural lesions. It is not a validated clinical decision rule, guideline, or stand-alone tool for treatment selection. As a future adjunct, CSF-based liquid biopsy may complement this pathway—particularly when tissue sampling is high-risk—by enabling detection of lymphoma-associated alterations in CSF ctDNA/cfDNA (e.g., MYD88 L265P and CD79B Y196), with CSF generally offering higher sensitivity than plasma in CNS settings [63]. Exploratory CSF biomarker approaches (including microRNA/exosome and cytokine/chemokine profiles) remain investigational and are not yet standardized for routine practice, requiring interpretation within the full clinical and radiological context [64,65].

Radiomics-based pipelines, coupled with machine-learning classifiers, may enable a more objective differentiation of dural lymphoma from meningioma and other dural mimickers by leveraging high-dimensional shape and texture features extracted from routine MRI (and, where available, functional sequences such as DWI/perfusion) and/or FDG PET. In primary CNS lymphoma, preliminary radiomics/ML studies have shown encouraging performance for non-invasive diagnostic classification, but translation to the dural compartment will require harmonized acquisition/segmentation workflows and rigorous external validation, ideally aligned with standardized feature definitions (e.g., IBSI) [66,67,68].

Within this framework, a structured set of clinico-radiological red flags operationalized as a Biopsy-First Score (Box 1) and early management principles (Box 2) are needed to ensure pathway reproducibility across neuroradiology, neurosurgery, and hemato-oncology. Box 1 translates “clinical context” into explicit, point-based criteria (Major = 2 points; Minor = 1 point) with pragmatic triage thresholds (0–1 low suspicion, 2–3 intermediate, ≥4 high). Box 2 translates this suspicion into operational decisions, clarifying when to prefer a low-morbidity “tissue-first” approach, when to reserve surgery for selected indications (decompression/debulking), and how to optimize the biopsy–staging–therapy sequence (including pragmatic considerations on corticosteroid timing). Figure 1 integrates Box 1 and Box 2 into an explicit decision pathway that makes clear when to deviate from conventional dural mass management and how to prioritize diagnostic and staging steps. Before proceeding to the initial branching of the diagnostic pathway, clinicians are encouraged to calculate the Biopsy-First Score (Box 1). This tool is intended as pragmatic decision support and does not replace clinical judgment; urgent decompression may take precedence (e.g., critical mass effect, medically refractory seizures, or neurological deterioration).

Evidence strength across the main decision nodes is heterogeneous and should be interpreted accordingly. Components supported by relatively stronger evidence include: (i) recognition of recurrent “meningioma-mimic” presentations in primary dural lymphoma series; (ii) prioritization of tissue diagnosis and comprehensive hematologic staging when lymphoma is a credible differential diagnosis; and (iii) post-biopsy staging/management principles aligned with established PCNSL/SCNSL recommendations (although not prospectively validated in a dural-lymphoma–specific algorithm). Components supported by more limited evidence include proposed score weighting and pragmatic thresholds in Box 1, quantitative imaging thresholds in dural-based lesions, and selected PET pitfalls (particularly in rarer or discordant scenarios), which are derived from smaller retrospective series, case reports, and expert synthesis and therefore require prospective multicenter validation.

Box 1 **Biopsy-First Score for “meningioma-like” dural lesions. Major criteria are assigned 2 points and minor criteria 1 point; proposed pragmatic thresholds (0–1 low suspicion; 2–3 intermediate suspicion; ≥4 high suspicion) guide triage toward standard management versus a biopsy-first pathway with expedited systemic staging when indicated. *Note:* This is a pragmatic triage tool synthesized from dural-lymphoma and dural-mimic imaging literature (not a formal guideline; not prospectively validated)**

Box 1. Biopsy-First Score for meningioma-like dural lesions: when to suspect a lymphomatous mimic and prioritize minimally invasive tissue diagnosis.A. Clinical red flags (context and time)Major: rapid symptom progression (days-weeks) or neurological deterioration atypical for meningioma. Major: hematologic history (known/recent lymphoma) and/or clinically significant immunosuppression. Major: skull-base phenotype (multiple cranial neuropathies) or suspicion of multicompartment disease. Minor: clinicoradiologic incongruence or disproportionate symptoms.B. Morphologic/structural red flags (CT/MRI, including bone)Major: atypical bone pattern: erosion/osteolysis or permeative change (rather than frank hyperostosis). Major: infiltrative behavior: blurred cortical interface or diffuse/multifocal pachymeningeal extension not fitting classic meningioma. Minor: disproportionate vasogenic edema relative to lesion size/implantation. Minor: en plaque or multifocal appearance that does not integrate with the clinical-radiologic context.C. Functional/metabolic red flags (multiparametric MRI and PET)Major: marked diffusion restriction (DWI/ADC) suggestive of hypercellularity in a non-convincing meningioma setting. Major: positive systemic staging (FDG-PET/CT or equivalent) suggesting secondary involvement/SCNSL. Major: receptor-imaging pitfall: SSTR-PET uptake compatible with meningioma but discordant with MRI and/or clinical context. Minor: perfusion/MRS parameters not aligned with typical meningioma (supportive, not decisive).Practical decision ruleBiopsy-First Score: assign 2 points to Major criteria and 1 point to Minor criteria, and interpret the total score using the proposed pragmatic thresholds: 0–1 = low suspicion (standard pathway), 2–3 = intermediate suspicion (biopsy-first approach favored if no decompressive indication is present), and ≥4 = high suspicion (biopsy-first approach with early systemic staging). This framework is intended to support early diagnostic stratification and multidisciplinary decision-making. Caveat: in case of critical mass effect, medically refractory seizures, or neurological deterioration, surgical debulking may precede full staging.Supporting sources (all cited in the main text): Karschnia et al., 2020 [1]; Lyndon et al. [44], 2019; Maireche et al., 2023 [45]; Juntikka et al., 2021 [51].

Box 2 **Pragmatic early management principles for suspected dural lymphoma, prioritizing timely biopsy and comprehensive hematologic staging before defining surgical strategy. *Interpretation:* Dedicated international guidelines for PDL are limited. The following principles are derived from PCNSL/SCNSL recommendations and apply when lymphoma is a credible differential diagnosis.**

Box 2. Guideline-consistent early management when CNS lymphoma is suspected: applied to meningioma-like dural lesions1. Prioritize tissue diagnosis (“biopsy-first”)Obtain targeted tissue sampling (stereotactic biopsy; alternatively, diagnostic mini-craniotomy when stereotaxy is impractical). CSF studies can be supportive when positive but have limited sensitivity and should not replace tissue diagnosis when an accessible lesion exists.2. Reserve resection/debulking for selected emergenciesRoutine gross-total resection is not a standard initial strategy for suspected CNS lymphoma. Consider decompression/debulking only when required for life-threatening mass effect/intracranial hypertension or when needed to obtain safe diagnostic tissue in a critical anatomical setting.3. If SCNSL is suspected, stage systemically and choose the safest biopsy sitePerform prompt systemic staging (FDG-PET/CT or equivalent total-body imaging) plus standard hematologic work-up. With concurrent systemic disease, diagnosis may be established from a systemic-site biopsy when CNS imaging is consistent after expert review; for isolated CNS relapse or atypical imaging, CNS biopsy is often required.4. Corticosteroids: avoid pre-biopsy if clinically feasibleWhen suspicion is high and the patient is stable, avoid or defer corticosteroids before biopsy due to potential reduction in diagnostic yield. If steroids are required (edema/intracranial hypertension/neurological deterioration), do not delay biopsy and document dose and duration.Practical one-line rule (for meningioma-like dural lesions)When red flags suggest a lymphomatous mimic and there is no primary decompressive indication, proceed with rapid minimally invasive tissue diagnosis and complete staging before planning extensive “meningioma-like” resection.Key sources (canonical publication years): Bobillo et al., 2023 [3]; Cwynarski et al., 2023 [7]; Hoang-Xuan et al., 2023 [10]; Ferreri et al., 2021 [19].

Beyond immediate clinical value, explicit criteria (Box 1 and Box 2) and algorithmic implementation (Figure 1) enable more robust evidence generation by harmonizing definitions and thresholds, building more comparable multicenter cohorts, and measuring practice-relevant endpoints: time to tissue diagnosis, rate of unnecessary extensive resections, completeness and appropriateness of staging, impact of pre-treatment steroids on diagnostic yield, and pathway safety. Without such minimum standards, the literature—dominated by retrospective series and reviews—remains intrinsically heterogeneous and difficult to translate into operational recommendations.

A priority research agenda should address three linked domains. First, the proposed framework (Box 1 and Box 2; Figure 1) requires prospective multicenter validation using pre-specified, practice-relevant endpoints, including time-to-diagnosis, rate of unnecessary extensive resections, staging appropriateness/completeness, treatment delay, and pathway-related safety outcomes. Second, imaging protocol harmonization is needed to improve reproducibility and comparability across centers, particularly for DWI/ADC acquisition and reporting, perfusion technique/metrics and post-processing, and PET acquisition/interpretive criteria (including management of discordant SSTR-PET scenarios). Third, standardized reporting templates for dural-based lesions should be developed to ensure consistent description of morphology, bone changes, diffusion/perfusion findings, PET patterns, and clinical red flags, thereby enabling more robust multicenter datasets and future consensus-building.

A realistic and methodologically coherent next step would be to develop multidisciplinary consensus recommendations (ideally via structured Delphi/expert panel processes with scientific society endorsement) formalizing: (i) Biopsy-First Score criteria and thresholds (Box 1), (ii) early management principles (Box 2), (iii) harmonized imaging acquisition and reporting requirements (including DWI/ADC, perfusion, and PET interpretive criteria) and standardized reporting templates for dural-based lesions, including predefined red-flag descriptors and bone-pattern terminology (iv) pathway quality indicators. These recommendations would provide a shared basis for multicenter validation of the proposed algorithm (Figure 1) and prospective studies, thereby laying the groundwork for future dedicated guidelines.

## 6. Limitations

This narrative review has methodological limitations. First, because it was not designed as a formal systematic review, it remains susceptible to selection bias despite a database search strategy and independent screening by two authors. In addition, language and publication biases may persist because we did not include grey literature, non-English studies, or contributions available only as abstracts.

The evidence base on dural and CNS lymphomas consists predominantly of retrospective studies, often with small cohorts and heterogeneous populations (PDL series versus cohorts derived from PCNSL/SCNSL), limiting comparability and generalizability—particularly for imaging findings and clinical outcomes. This limitation is particularly relevant for imaging biomarkers, because heterogeneity in acquisition protocols, post-processing methods, region-of-interest strategies, and reporting terminology further reduces cross-study comparability and may exaggerate the apparent specificity of isolated imaging features.

Importantly, the proposed algorithm and Biopsy-First Score are pragmatic, hypothesis-generating triage frameworks rather than validated decision rules or guidelines, and they should not be used as stand-alone tools. In addition, the level of evidence is not uniform across algorithm components: some points (e.g., biopsy/staging prioritization when lymphoma is suspected) are supported by recurrent patterns across retrospective series and by guideline-consistent principles extrapolated from PCNSL/SCNSL, whereas other components (e.g., selected PET pitfalls, quantitative imaging or biomarker thresholds in rarer scenarios) are supported by smaller series or case reports and remain exploratory.

To improve transparency, we performed a structured methodological quality appraisal of the major included studies (Supplementary Table S2). However, this appraisal was used to contextualize and interpret the evidence rather than as an exclusion framework, because the available literature is sparse and largely retrospective. Accordingly, residual risks of selection bias, ascertainment bias, inconsistent imaging/staging reporting, and incomplete outcome follow-up remain important constraints on the strength and generalizability of the proposed framework.

Eventually, the Biopsy-First Score represents a pragmatic, hypothesis-generating triage framework rather than a validated clinical decision rule or practice guideline. Its inter-rater reproducibility and performance require formal evaluation across centers and imaging workflows to determine generalizability and robustness.

## 7. Conclusions

Extra-axial dural lesions are frequently evaluated with insufficient radiological rigor, creating a risk of premature diagnostic impact that may inappropriately shape clinical management and expose patients to incorrect treatments. In cases with diagnostic uncertainty—particularly in the absence of significant mass effect or medically refractory seizures—direct referral to craniotomy rather than a biopsy procedure may entail substantially greater procedural risk than a minimally invasive diagnostic approach.

This issue is especially relevant when imaging raises suspicion for dural lymphoma: in such contexts, a biopsy-first pathway may prevent unnecessary resections and facilitate timely initiation of disease-directed therapy. Despite increasing recognition of pachymeningeal lymphomatous involvement, dedicated guidelines for dural lymphoma management are still lacking, and current practice often relies on evidence extrapolated from parenchymal primary CNS lymphoma.

Against this backdrop, the present narrative review aims to provide above all, a critical and as comprehensive as possible, synthesis of the radiological characteristics of extra-axial dural lesions, with particular attention to entities with overlapping phenotypes. The overarching goal is to support a more accurate and efficient diagnostic pathway and better-informed treatment decisions through a structured methodological approach and a practical neuroradiological guide.

## Figures and Tables

**Table 1:** Major published case series of dural lymphoma: A. Primary dural lymphoma/intracranial marginal zone lymphoma (meningioma-like) series; B. Secondary CNS lymphoma with dural/pachymeningeal involvement (SCNSL/secondary dural involvement).

Study (Year)	Design/Setting	Patients, n	Histology/Cohort	Treatment/Management	Key Outcomes/Notes
A. Primary dural lymphoma/intracranial marginal zone lymphoma (meningioma-like) series					
Karschnia (2020) [1]	Bi-institutional neuro-oncology cohort (PDL within PCNSL denominator)	20	Mixed: DLBCL 10/20, MZL 6/20, follicular 2/20, other 2/20	Individualized: surgery/biopsy + adjuvant RT and/or systemic therapy (including CNS-directed regimens for aggressive histology)	Best response evaluable (*n* = 16): response 88%, CR 69%; median follow-up 44 months; 3 deaths from progression
de la Fuente (2017) [4]	Multi-institution experience (MSKCC + Univ. Miami): dural marginal zone lymphoma	26	Predominantly MZL/EMZL (dural)	Biopsy/surgery common for diagnosis; focal RT frequently used; systemic therapy in selected	Upfront resection for presumed meningioma: 17/26 (65%). Evaluable (*n* = 23): ORR 100%, CR 22/23; 3-year PFS 89%, 5-year PFS 76%, 5-year OS 92%; relapses reported (local and systemic).
Venkataraman (2011) [5]	Pathology-anchored series of dural MZL (IgG4 link explored)	32	MZL involving meningeal dura; IgG4-positive plasma cells in a subset	Treatment heterogeneous in reported cohort	For cases with follow-up available (17): 16/17 alive, disease-free (4–124 months; median ~19.5 months)
Iwamoto (2006) [11]	PDL treatment/outcome series	8	Low-grade MZL in all cases	RT in all; chemotherapy in 3 (adjuvant/combined)	Complete response in all; no local failures; systemic relapse described in 3 (late extracranial disease)
Tu et al. (2005) [12]	Clinicopathologic/genetic series: intracranial MZL mimicking meningioma.	15 (14/15 dural-based masses)	Marginal zone B-cell lymphoma; plasmacytoid differentiation common; trisomy 3 frequent.	Surgery/biopsy for diagnosis; RT and/or chemotherapy (as reported).	For cases with follow-up (10): no evidence of disease at 1–7.6 years.
Sunderland et al. (2020) [13]	International multicenter retrospective analysis: EMZL with CNS/dural involvement.	26	EMZL with histologically confirmed CNS/dural involvement; primary CNS MZL (*n* = 15) vs. secondary CNS MZL (*n* = 11).	Heterogeneous; surgery/biopsy + RT and/or systemic therapy.	Dura most common site (50%); 2-year PFS 59%, OS 80%; secondary CNS MZL: 2-year PFS 54%, OS 58%.
B. Secondary CNS lymphoma with dural/pachymeningeal involvement (SCNSL/secondary dural involvement)					
El-Galaly et al. (2018) [14]	Large international retrospective cohort (registries/databases; immunochemotherapy era).	291 patients with SCNS involvement by DLBCL.	DLBCL with secondary CNS involvement during/after first-line; CNS sites categorized (parenchymal, leptomeningeal, both) but dural not separated in abstract.	Curative/intensive therapy in a subset (e.g., HD-MTX- or platinum-containing regimens); variable use of rituximab and HDC/ASCT in responders.	Median post-SCNS OS 3.9 months; 2-year OS 20% (95% CI 15–25). In patients ≤60 with PS 0–1 treated with HD-MTX-based regimens for isolated parenchymal SCNS: 2-year OS 62% (95% CI 36–80).
Hollender et al. (2000) [15]	Single-center retrospective cohort (systemic NHL with CNS involvement).	140	Systemic NHL with CNS involvement at diagnosis/relapse/progression.	Chemo +/− RT; HDT used in a small subset (as reported).	Median survival after CNS diagnosis 2.6 months; better prognosis when CNS involvement at initial diagnosis; paresis predicted survival.
Jahnke et al. (2006) [16]	Single-center retrospective prognostic-factor study.	43	NHL with secondary CNS involvement.	IT chemotherapy 77%; systemic chemotherapy 58%; RT 37%.	Median survival 5 months; low LDH and response to CNS therapy associated with improved survival.
Ferreri et al. (2015) [17]	Multicenter phase II trial: HD-MTX/Ara-C -> R-HDS -> ASCT (+ IT liposomal Ara-C).	38	Aggressive B-cell lymphoma with secondary CNS involvement (diagnosis or relapse).	HD-MTX + cytarabine; rituximab; R-HDS; ASCT in eligible responders.	CR 63%; 2-year EFS 50% +/− 8%; 5-year OS 41% +/− 8% overall and 68% +/− 11% in transplanted; 4 treatment-related deaths.
Malikova et al. (2018) [18]	Retrospective imaging series: morphological MRI patterns in SCNSL.	21	SCNSL (histologically verified); steroids at MRI in 12/21.	Not a treatment study (diagnostic imaging focus).	Parenchymal lesions 86%; combined parenchymal + meningeal involvement 48%; diffusion restriction in 78% of parenchymal lesions.
Ferreri et al. (2021) [19]	MARIETTA trial (international, single-arm phase II): MATRix-RICE + ASCT.	79 enrolled; 75 assessable	DLBCL with secondary CNS involvement.	MATRix ×3 -> RICE ×3; carmustine/thiotepa-conditioned ASCT in responders.	1-year PFS 58%; 2-year PFS 46% overall and 83% in transplanted; 2-year OS 46% overall and 83% in transplanted.
Habringer et al. (2024) [20]	International prospective registry (SCNSL-R; NCT05114330).	279 enrolled; 243 analyzed	SCNSL of all entities (DLBCL 68%); synchronous 20% vs. metachronous 80%.	Real-world induction regimens; HDT-ASCT consolidation in responders (PR or better) associated with OS benefit.	Median OS 17.2 months; longer OS in synchronous cohort (60.6 months) vs. metachronous (11.4 months); HDT-ASCT in responders: adjusted HR 0.47 for OS.
Spectre et al. (2005) [21]	Retrospective single-center series (indolent B-cell lymphomas with CNS involvement).	7 patients.	Indolent B-cell lymphomas with CNS involvement; parenchymal (2), leptomeningeal (3), both (2); systemic disease in all (bone marrow in all but one).	Systemic + intra-CSF chemotherapy in 6/7; radiotherapy in 2.	CNS response in 5 patients; survival 1–9 years for treated patients (median 2 years); 3 died of CNS disease.

*Abbreviations: PDL = primary dural lymphoma; MZL = marginal zone lymphoma; EMZL = extranodal marginal zone lymphoma; DLBCL = diffuse large B-cell lymphoma; SCNSL = secondary CNS lymphoma; CNS = central nervous system; RT = radiotherapy; HD-MTX = high-dose methotrexate; Ara-C = cytarabine; IT = intrathecal; ASCT = autologous stem-cell transplantation; HDT-ASCT = high-dose therapy with ASCT; CR = complete response; PR = partial response; EFS = event-free survival; PFS = progression-free survival; OS = overall survival; R-HDS = rituximab plus high-dose sequential chemoimmunotherapy; MATRix = rituximab, methotrexate, cytarabine, thiotepa; RICE = rituximab, ifosfamide, carboplatin, etoposide.*

**Table 2:** Typical clinico-radiological profiles across the four operational clusters of dural lymphoma. The table summarizes, for each cluster, the usual clinical setting, presenting phenotype, key discriminator(s) used in day-to-day differential diagnosis, initial management priorities, and typical outcome patterns and follow-up implications. The schema is intended as a pragmatic aid to multidisciplinary decision-making and does not replace tissue diagnosis. PDL = primary dural lymphoma; EMZL = extranodal marginal zone lymphoma; MALT = mucosa-associated lymphoid tissue; DLBCL = diffuse large B-cell lymphoma; SCNSL = secondary central nervous system lymphoma; CNS = central nervous system; HD-MTX = high-dose methotrexate.

Cluster	Setting	Typical Profile	Key Discriminator	Initial Management	Prognosis/Follow-Up
1A	Indolent PDL (extranodal marginal zone/MALT type)	Middle-aged (often female), immunocompetent; subacute mass-effect symptoms. Dural mass along convexity/falx/tentorium or skull base; frequently interpreted as meningioma.	Disease confined to dura at diagnosis; systemic staging negative (operational definition of PDL).	Tissue diagnosis (biopsy or limited resection if decompression needed); focal radiotherapy as indicated by location and symptom burden.	Excellent local control; prolonged follow-up to detect rare late extracranial relapse.
1B	PDL, non-EMZL/MALT (mainly primary dural diffuse large B-cell lymphoma; rare other histotypes)	Meningioma-like phenotype with rapid progression (weeks) and neurological deterioration. Skull base/cavernous sinus neuropathies; spinal dural/epidural compression may occur.	Aggressive clinical course with negative systemic staging; higher likelihood of requiring central nervous system-directed systemic therapy.	Central nervous system-directed systemic therapy (high-dose methotrexate-based regimens) ± focal radiotherapy; surgery mainly for diagnosis and/or urgent decompression.	Less favorable and more heterogeneous than Cluster 1A; outcome driven by biology and treatment intensity.
2A	Dural involvement in secondary central nervous system lymphoma from aggressive systemic lymphomas (predominantly diffuse large B-cell lymphoma)	Dural disease is usually not isolated; multi-compartment central nervous system involvement (parenchymal, leptomeningeal, cranial nerve/root). Often early in high-risk systemic subsets.	Systemic lymphoma present; systemic staging positive by definition (secondary involvement).	Intensive central nervous system-directed regimens (high-dose methotrexate and cytarabine backbones); consider intrathecal therapy and autologous transplant in selected patients.	Overall worse outcomes than PDL; prognosis driven by systemic risk profile, central nervous system burden, and feasibility of intensive therapy.
2B	Dural involvement in disseminated indolent systemic lymphomas	Rare; single or multiple meningioma-like dural masses or diffuse pachymeningeal thickening (including en plaque patterns). Mixed leptomeningeal/parenchymal patterns may occur.	Imaging overlaps with Cluster 1A; practical discriminator is evidence of systemic indolent lymphoma on staging.	Integrate neuroimaging with systemic staging; tailor therapy to histotype, systemic burden, and pattern of central nervous system involvement.	Variable; depends on underlying indolent lymphoma biology and extent/pattern of central nervous system involvement.

## Data Availability

No new data were created or analyzed in this study. Data sharing is not applicable to this article.

## Supplementary Materials

The following supporting information can be downloaded at: https://www.mdpi.com/article/10.3390/cancers18152369/s1, Table S1: Example of PubMed/MEDLINE search string; Table S2: Simplified structured methodological quality appraisal of the major clinical studies included in the narrative synthesis.

## Abbreviations

The following abbreviations are used in this manuscript:

18F-FDG	fluorine-18 fluorodeoxyglucose
3D	three-dimensional
68Ga-DOTATATE/DOTATOC	gallium-68 somatostatin receptor tracers
ABC	activated B-cell-like
ADC	apparent diffusion coefficient
Ara-C	cytarabine
ASCT	autologous stem cell transplantation
BCL6	B-cell lymphoma 6
BR	bendamustine–rituximab
BTK	Bruton tyrosine kinase
CD	cluster of differentiation
CI	confidence interval
CNS	central nervous system
CR	complete response
CSF	cerebrospinal fluid
ctDNA	circulating tumor DNA
cfDNA	cell-free DNA
CT	computed tomography
DAAs	direct-acting antivirals
DCE	dynamic contrast-enhanced (MRI)
DLBCL	diffuse large B-cell lymphoma
DSC	dynamic susceptibility contrast (MRI)
DWI	diffusion-weighted imaging
EFS	event-free survival
EHA-ESMO	European Hematology Association–European Society for Medical Oncology
EMZL	extranodal marginal zone lymphoma
FDG	fluorodeoxyglucose
FDG-PET/CT	fluorodeoxyglucose positron emission tomography/computed tomography
FLAIR	fluid-attenuated inversion recovery
GCB	germinal center B-cell-like
HDC	high-dose chemotherapy
HDC-ASCT	high-dose chemotherapy followed by autologous stem cell transplantation
HD-MTX	high-dose methotrexate
HDS	high-dose sequential chemoimmunotherapy
HDT	high-dose therapy
HDT-ASCT	high-dose therapy with autologous stem cell transplantation
HCV	hepatitis C virus
HCV-RNA	hepatitis C virus ribonucleic acid
HR	hazard ratio
IBSI	Image Biomarker Standardisation Initiative
IgG4	immunoglobulin G4
IgG4-RD	IgG4-related disease
IRF4	interferon regulatory factor 4
IT	intrathecal
Ki-67	Ki-67 proliferation index
LDH	lactate dehydrogenase
MALT	mucosa-associated lymphoid tissue
MARIETTA	MATRix-RICE and autologous stem cell transplantation trial acronym
MATRix	rituximab, methotrexate, cytarabine, thiotepa (regimen)
MCD	molecular class MCD (LymphGen)
MeSH	Medical Subject Headings
ML	machine learning
MRI	magnetic resonance imaging
MRS	magnetic resonance spectroscopy
MSKCC	Memorial Sloan Kettering Cancer Center
MUM1	multiple myeloma oncogene 1
MYD88	myeloid differentiation primary response 88
MZL	marginal zone lymphoma
NHL	non-Hodgkin lymphoma
non-GCB	non–germinal center B-cell-like
ORR	objective response rate
OS	overall survival
PAX5	paired box 5
PCR	polymerase chain reaction
PCNSL	primary central nervous system lymphoma
PD-DLBCL	primary dural diffuse large B-cell lymphoma
PDL	primary dural lymphoma
PET	positron emission tomography
PET/CT	positron emission tomography/computed tomography
PET/MRI	positron emission tomography/magnetic resonance imaging
PFS	progression-free survival
PR	partial response
rCBV	relative cerebral blood volume
R-CHOP	rituximab, cyclophosphamide, doxorubicin, vincristine, prednisone
R-HDS	rituximab plus high-dose sequential chemoimmunotherapy
RICE	rituximab, ifosfamide, carboplatin, etoposide
R-MBVP	rituximab, methotrexate, carmustine, etoposide, prednisone
R-MPV	rituximab, methotrexate, procarbazine, vincristine
R-MT	rituximab–methotrexate
RT	radiotherapy
SCNSL	secondary central nervous system lymphoma
SCNSL-R	Secondary CNS Lymphoma Registry
SSTR	somatostatin receptor
SSTR-PET	somatostatin receptor positron emission tomography
SWI	susceptibility-weighted imaging
T1	T1-weighted (MRI)
T2	T2-weighted (MRI)
T2*	T2*-weighted (MRI)
WHO	World Health Organization
WHO CNS5	WHO Classification of Tumors of the Central Nervous System, 5th edition
WHO-HAEM5	WHO Classification of Hematolymphoid Tumors, 5th edition
