# Supplementary material for: Dural Lymphomas Unmasked: A Narrative Review of Extra-Axial Mimics and a Pragmatic Diagnostic Decision Algorithm
Source: Cancers (Basel). 2026 Jul 23;18(15):2369. doi: 10.3390/cancers18152369 (PMC13465272; doi:10.3390/cancers18152369)
Supplement: Supplementary file 1 [file cancers-18-02369-s001.zip › Supplement (Table S1).pdf]

Table S1: Example of PubMed/MEDLINE search string

```
(
 (
  "Dura Mater"[Mesh]
  OR dura[tiab]
  OR dural[tiab]
  OR pachymening*[tiab]
  OR "dural-based"[tiab]
  OR "dura-based"[tiab]
  OR "extra-axial"[tiab]
 )
 AND
 (
  "Lymphoma"[Mesh]
  OR lymphoma*[tiab]
  OR "dural lymphoma"[tiab]
  OR "primary dural lymphoma"[tiab]
  OR "primary meningeal lymphoma"[tiab]
  OR "pachymeningeal lymphoma"[tiab]
  OR "central nervous system lymphoma"[tiab]
  OR "CNS lymphoma"[tiab]
  OR "secondary CNS lymphoma"[tiab]
  OR SCNSL[tiab]
  OR "marginal zone lymphoma"[tiab]
  OR "extranodal marginal zone lymphoma"[tiab]
  OR MALT[tiab]
  OR "diffuse large B-cell lymphoma"[tiab]
  OR DLBCL[tiab]
 )
)
AND
(
 meningioma*[tiab]
 OR mimic*[tiab]
 OR "differential diagnos*"[tiab]
 OR imaging[tiab]
 OR radiolog*[tiab]
 OR "magnetic resonance imaging"[tiab]
 OR MRI[tiab]
 OR "computed tomography"[tiab]
 OR "positron emission tomography"[tiab]
 OR PET[tiab]
 OR "PET/CT"[tiab]
 OR "FDG-PET"[tiab]
 OR biopsy[tiab]
 OR resection[tiab]
 OR surgery[tiab]
)
```
