# Supplementary material for: Dural Lymphomas Unmasked: A Narrative Review of Extra-Axial Mimics and a Pragmatic Diagnostic Decision Algorithm
Source: Cancers (Basel). 2026 Jul 23;18(15):2369. doi: 10.3390/cancers18152369 (PMC13465272; doi:10.3390/cancers18152369)
Supplement: Supplementary file 1 [file cancers-18-02369-s001.zip › Supplementary_Table_S2.pdf]

Supplementary Table S2. Simplified structured methodological quality appraisal of the major clinical studies included in the narrative synthesis.

Appraisal was used for interpretive weighting and transparency (not as an exclusion criterion). Ratings reflect reporting completeness and major bias concerns in heterogeneous retrospective evidence and selected interventional studies.

Group	Study (year)	Design	Role in review	Cohort / denominator	Diagnostic / imaging / staging reporting	Treatment / outcomes / follow-up reporting	Overall interpretive weight	Main limitation(s)
PDL	Tu et al. (2005)	Clinicopathologic/genetic series	PDL mimicry / pathology context	Y	P	P	Moderate	Pathology-focused; imaging/staging and follow-up not uniform
PDL	Iwamoto (2006)	Retrospective PDL series	PDL outcomes / treatment context	Y	P	P	Moderate	Small sample size (n=8); retrospective design
PDL	Venkataraman (2011)	Pathology-anchored dural MZL series	PDL pathology and diagnostic spectrum	Y	P	P	Moderate	Strong pathology detail; heterogeneous treatment/follow-up reporting
PDL	de la Fuente (2017)	Multi-institution retrospective cohort	Core PDL treatment/outcome evidence	Y	P	Y	High (PDL)	Retrospective multicenter heterogeneity
PDL	Sunderland et al. (2020)	International multicenter retrospective cohort	Contextual CNS/dural MZL evidence	Y	P	P	Moderate (contextual)	Mixed primary/secondary CNS MZL; not purely PDL
PDL	Karschnia (2020)	Bi-institutional retrospective neuro-oncology cohort	PDL within broader CNS lymphoma framework	Y	P	P	Moderate	Referral/selection bias possible; outcomes evaluable in subset
SCNSL	Hollender et al. (2000)	Single-center retrospective cohort	Historical prognosis context (SCNSL)	Y	N/P	P	Low-Moderate (contextual)	Historical cohort; dural involvement not isolated
SCNSL	Spectre et al. (2005)	Small retrospective series	Indolent lymphoma with CNS involvement context	Y	P	P	Low (contextual)	Very small sample (n=7); mixed CNS compartments
SCNSL	Jahnke et al. (2006)	Retrospective prognostic study	Prognostic context in SCNSL	Y	N/P	Y	Moderate (prognostic context)	Limited dural-specific granularity and imaging detail

SCNSL	Ferreri et al. (2015)	Multicenter phase II trial	SCNSL treatment pathway context	Y	P/Y	Y	High (management context)	Not dural-specific; designed for treatment efficacy
SCNSL	El-Galaly et al. (2018)	Large international retrospective cohort	SCNSL epidemiology/outcome context	Y	P	Y	High (contextual SCNSL)	CNS compartments reported, but dural subset not separated
SCNSL	Malikova et al. (2018)	Retrospective imaging series	MRI pattern context (SCNSL)	Y	Y	NA	Moderate (imaging context)	Imaging-focused study; not for treatment/outcome inference
SCNSL	Ferreri et al. (2021)	International phase II (MARIETTA)	SCNSL intensive treatment framework	Y	P/Y	Y	High (management context)	Not dural-specific ascertainment
SCNSL	Habringer et al. (2024)	International prospective registry	Contemporary real-world SCNSL management/outcomes	Y	P	Y	High (contextual SCNSL)	Registry not designed for dural-specific analyses

Legend: Y = adequately reported / low concern; P = partially reported / heterogeneous reporting; N = not clearly reported or not separable; NA = not applicable to the primary study aim/design.

Supplementary Table S2. Simplified structured methodological quality appraisal of the major clinical studies included in the narrative synthesis.
Appraisal was used for interpretive weighting and transparency (not as an exclusion criterion). Ratings reflect reporting completeness and major bias concerns in heterogeneous retrospective evidence and selected interventional studies.
